# The Use of JAK Inhibitors in Elderly Patients with Moderate-to-Severe Atopic Dermatitis: A Narrative Review of Clinical and Real-World Evidence

**DOI:** 10.3390/jcm14176327

**Published:** 2025-09-08

**Authors:** Giuseppe Lauletta, Daniele Cecere, Luca Potestio, Francesca di Vico, Cataldo Patruno, Maddalena Napolitano

**Affiliations:** 1 Section of Dermatology, Department of Clinical Medicine and Surgery, University of Naples Federico II, 80131 Naples, Italy; 2 Department of Medicine and Health Sciences “Vincenzo Tiberio”, University of Molise, 86100 Campobasso, Italy

**Keywords:** atopic dermatitis, elderly patients, JAK inhibitors, abrocitinib, baricitinib, upadacitinib

## Abstract

**Background:** Atopic dermatitis (AD) in elderly patients presents unique clinical challenges due to comorbidities, polypharmacy, and an increased risk of adverse events. Janus kinase inhibitors (JAKis) have emerged as effective oral treatments, but limited data are available for their use in older adults. **Methods:** A narrative review was conducted through a comprehensive search of PubMed/MEDLINE, Ovid, Scopus, Embase, Cochrane Library, Web of Science, and Google Scholar up to July 2025. Only English-language studies evaluating abrocitinib, baricitinib, or upadacitinib in elderly patients (≥65 years) with moderate-to-severe AD were included. **Results:** RCTs enrolled few elderly patients and lacked dedicated subgroup analyses. Abrocitinib showed similar efficacy in older and younger adults, but higher rates of adverse events, especially at the 200 mg dose. Real-world studies, mainly on upadacitinib, demonstrated rapid and sustained clinical improvements, including in difficult-to-treat areas. Safety outcomes were generally favorable; most adverse events were mild and manageable. Herpes zoster and hematological changes were more frequent in elderly patients, while serious cardiovascular events were rare. **Conclusions:** JAK inhibitors are effective therapeutic options for elderly patients with moderate-to-severe AD. Careful patient selection, dose tailoring, and close monitoring are essential. Further age-specific RCTs and long-term real-world data are needed to guide clinical practice.

## 1. Introduction

Atopic dermatitis (AD) is a chronic inflammatory condition of the skin, typically marked by pruritus and eczematous lesions, which commonly affect areas such as the face, neck, and limb flexures depending on age [1,2]. Although previously considered primarily a pediatric condition, recent evidence indicates that AD can occur or persist in adults, including those of advanced age [3]. Epidemiological data from a UK-based cohort study of 9,154,936 individuals aged 0–99 years, drawn from The Health Improvement Network (THIN) between 1994 and 2013, estimate a prevalence of 7.0% to 9.3% among those aged 75–99 years [4]. In older adults, AD may present with non-standard clinical features such as generalized eczema, nummular eczema, or prurigo nodularis, which may make diagnosis more challenging [5,6]. Additionally, moderate-to-severe forms appear to be more prevalent in this demographic compared to younger patients [4]. Managing AD in the elderly poses distinct challenges due to common comorbidities, polypharmacy, and the potential for adverse drug interactions. These factors often limit the use of conventional systemic therapies like cyclosporine, methotrexate, azathioprine or mycophenolate mofetil [7,8]. Although biologic agents such as dupilumab, tralokinumab, and lebrikizumab (not yet approved in all global regulatory regions) demonstrate favorable safety profiles [9,10,11,12,13], a subset of patients either does not respond adequately or cannot tolerate these treatments due to specific contraindications (e.g., hypersensitivity, injection-related issues) [14]. Janus kinase inhibitors (JAKis) have emerged as promising oral alternatives for AD management. Approved agents include abrocitinib (a selective JAK1 inhibitor), baricitinib (inhibiting JAK1 and JAK2), and upadacitinib (a selective and reversible JAK1 inhibitor) [8]. Nevertheless, concerns about their safety have been raised, particularly for older patients. The European Medicines Agency (EMA) advises restricting JAKis use in those aged 65 and above unless other treatment options are unsuitable [15]. This caution is grounded in the known risks associated with JAKis, including serious infections, malignancies, and cardiovascular complications [16,17]. Moreover, elderly populations are often underrepresented in pivotal randomized clinical trials (RCTs). For example, participants over 75 years were excluded from major studies evaluating abrocitinib (JADE MONO-1, JADE MONO-2), baricitinib (BREEZE-AD), and upadacitinib (MEASURE UP 1, MEASURE UP 2, AD UP) [18]. As such, there is a growing need for real-world evidence to better assess the utility and safety of these therapies in older adults. The present review aims to address this gap by evaluating the effectiveness and safety of JAKis (abrocitinib, baricitinib, and upadacitinib) in elderly patients with moderate-to-severe AD.

## 2. Materials and Methods

**Figure 1:**
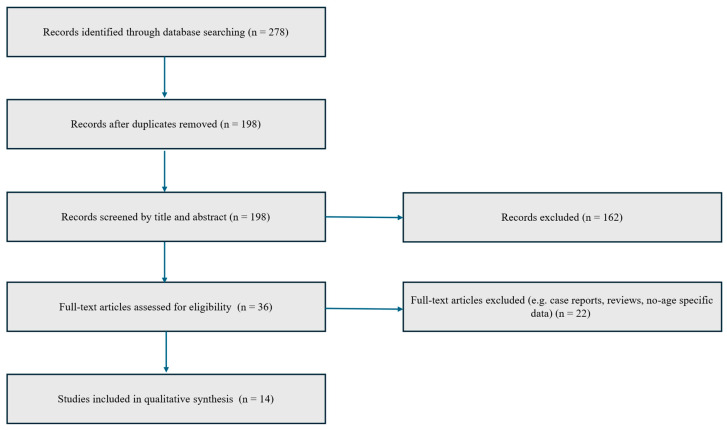
PRISMA-style flow diagram illustrating the study selection process for inclusion in the narrative review.

A comprehensive narrative review was conducted by systematically exploring the medical literature available up to 10 July 2025, through databases including PubMed/MEDLINE, Ovid, Scopus, Embase, Cochrane Library, Web of Science, and Google Scholar. The research focused on the pharmacological agents abrocitinib, baricitinib, and upadacitinib, utilizing both Medical Subject Headings (MeSH) and relevant medical terminology. Search queries combined terms such as “abrocitinib”, “baricitinib”, and “upadacitinib” with keywords like “atopic dermatitis”, “pruritus”, “clinical trial”, and “real-life studies”. The strategy included scanning all parts of the articles—titles, abstracts, keywords, and full texts—to ensure thorough coverage. Reference lists of pertinent articles were also reviewed to identify additional sources that might have been missed during the initial search. To reduce selection bias, two independent authors (G.L. and M.N.) performed the screening of retrieved titles and abstracts. Any discrepancies were resolved through discussion and consensus. The inclusion criteria were as follows: (1) studies evaluating JAK inhibitors in adult patients with moderate-to-severe AD; (2) studies reporting data specifically for patients aged ≥60 years or including them as a defined subgroup; (3) randomized controlled trials or real-world studies with original data. The exclusion criteria included: (1) case reports, reviews, editorials, and conference abstracts; (2) studies without accessible full text; (3) studies not reporting any age-specific data or results. Through this search strategy, a total of 278 potentially relevant articles were identified. After removal of duplicates and application of eligibility criteria, 14 studies were included in the present review. A simplified PRISMA-style flow diagram illustrating the study selection process is provided (Figure 1). In all included studies, dosing regimens were consistent with approved schedules for moderate-to-severe AD. Specifically, abrocitinib was administered at 100 mg or 200 mg once daily, upadacitinib at 15 mg or 30 mg once daily, and baricitinib at 2 mg or 4 mg once daily.

Studies were then grouped by individual JAK inhibitor (abrocitinib, baricitinib, upadacitinib), and further stratified according to study design (RCTs vs. real-world evidence), with a dedicated synthesis of results from elderly subpopulations when data were available. It is important to note that the data discussed herein are based entirely on previously published research.

## 3. Results

### 3.1. Randomized Clinical Trials (RCTs)

#### 3.1.1. Upadacitinib

In the long-term extension study of the Measure Up 1 and 2 phase 3 trials, the authors evaluated the sustained efficacy and safety of upadacitinib up to 160 weeks in patients aged 12 to 75 years [19]. Although individuals aged ≥65 years were eligible and likely included based on the age range, the total number of elderly participants was not specified [19]. Moreover, no subgroup analyses were performed to specifically assess clinical outcomes in this population. As a result, while the study demonstrated durable improvements in skin clearance and symptom control across the overall cohort, the long-term efficacy and safety profile of upadacitinib in older adults remains undefined within this trial [19]. The same applies to the trial evaluating upadacitinib in combination with topical corticosteroids, in which older adults (>65 years) were included but represented a small proportion of the total study population, 36 individuals (approximately 4% of the full cohort) [20]. However, no separate efficacy or safety outcomes were reported for this subgroup. Although this study demonstrates rapid and sustained improvements in pruritus and quality of life across the overall cohort, it does not offer definitive evidence regarding the therapeutic profile of upadacitinib in elderly patients [20].

#### 3.1.2. Baricitinib

A phase 3 North American study evaluated baricitinib 1 mg and 2 mg in adults with moderate-to-severe AD. Although the trial did not include a specific subgroup analysis for patients >65 years, it provides relevant safety insights [21]. The baricitinib 2 mg group showed significant improvement in Eczema Area Severity Index (EASI), Validated Investigators Global Assessment (vIGA)-AD 0/1, itch, sleep disturbance, and quality of life (QoL) by W16 [21]. Importantly, no serious adverse events (SAEs) such as malignancies, thromboembolic events, or major cardiovascular events (MACEs) were observed [21]. These findings indicate a short-term safety profile for baricitinib in older adults; however, additional age-stratified data are needed [21].

#### 3.1.3. Abrocitinib

Across the pivotal abrocitinib RCTs (JADE MONO-1, MONO-2, COMPARE, REGIMEN, and EXTEND), 146 individuals aged 65 years or older were included, though they represented only a small fraction of the study populations (N = 146, 4.62%) [22].

Despite the limited number of elderly participants, their clinical response to abrocitinib appeared broadly consistent with that of younger adults [22]. Improvements in pruritus severity and skin clearance (as measured by endpoints such as EASI-75 and IGA) were observed in older patients at levels comparable to the general trial population. However, due to the small sample size, these findings should be interpreted as exploratory [22].

Concerning safety, older adults exhibited a trend toward increased incidence of several treatment-emergent adverse events (TEAEs) compared to younger age groups. Notably, the incidence rates of SAEs and treatment discontinuations due to TEAEs were higher in older patients, especially those receiving the 200 mg dose [22]. Among patients aged ≥65 years treated with abrocitinib 200 mg, the incidence of SAEs reached 19.18 events per 100 patient-years, and that of discontinuations was 32.34 per 100 patient-years. These rates were somewhat lower with the 100 mg dose (15.62 and 16.03 per 100 patient-years, respectively), suggesting a more favorable safety profile at the lower dosage in this age group [22].

Infectious complications, particularly herpes zoster, were also more frequent in the elderly. Herpes zoster infections occurred with an incidence of 10.03 per 100 patient-years in patients receiving abrocitinib 200 mg and 5.61 per 100 patient-years in those on 100 mg [22]. Serious infections followed a similar pattern, with incidence rates of 6.27 and 3.97 per 100 patient-years for the 200 mg and 100 mg doses, respectively. Although the majority of Herpes Zoster cases were non-serious and limited to dermatomal involvement, these findings underscore the need for vaccination and careful monitoring in older patients [22].

Age-related hematologic abnormalities were also observed, particularly in the 200 mg group. Thrombocytopenia (platelet count <75,000/mm^3^) and lymphopenia (lymphocyte count <500/mm^3^) occurred exclusively in older patients receiving the higher dose, with incidence rates of 3.17 and 6.29 per 100 patient-years, respectively. These laboratory changes were not observed in elderly patients treated with the 100 mg dose [22].

Consistent with expectations, the incidence of malignancies—including non-melanoma skin cancers (NMSC) and other tumors—was observed to be numerically greater among patients aged 65 years and older in both dosage groups. For NMSC, incidence rates were 2.13 and 2.74 per 100 patient-years for the 200 mg and 100 mg doses, respectively, while rates for other malignancies were 3.20 and 1.32 per 100 patient-years. Notably, all skin cancers occurred in patients with a history of smoking [22].

Finally, cardiovascular and thromboembolic events, including MACEs and venous thromboembolism (VTE), were more frequently reported in older patients, particularly in those treated with 100 mg abrocitinib. In this subgroup, MACEs occurred at a rate of 2.64 per 100 patient-years, while the corresponding rate for VTE was also 2.64 per 100 patient-years [22]. Interestingly, more MACEs were observed in the 100 mg group compared to the 200 mg group in the pooled analysis. This paradoxical finding is likely attributable to baseline differences in cardiovascular risk profiles or selection bias rather than a true dose-related effect, as patients deemed frailer or at higher risk may have preferentially received the lower dose. However, given the limited number of elderly patients included in the pooled analysis (n = 146), these findings should be interpreted with caution, particularly regarding rare events such as malignancies or MACEs. 

Data from clinical trials on elderly patients with moderate-to-severe AD treated with JAK inhibitors have been summarized in Table 1.

### 3.2. Real Life Studies

#### 3.2.1. Upadacitinib

In a recent multicenter retrospective study conducted across six Spanish hospitals, Melgosa Ramos et al. assessed the real-world effectiveness and safety of upadacitinib in adults aged over 50 years with moderate-to-severe AD [23]. The study included 38 patients, with a mean age of 63.9 years. Upadacitinib treatment (administered at either 15 mg or 30 mg daily) resulted in significant clinical improvement across multiple disease domains [23]. By week (W) 16, 65.8% of patients achieved Minimal Disease Activity (MDA), as defined by composite endpoints integrating EASI, IGA, p-NRS, Dermatology Life Quality Index (DLQI), and Body Surface Area (BSA) scores [23]. Among the 13 patients with long-term follow-up data at W52, 84.6% maintained MDA, indicating sustained disease control over time. These outcomes were observed across AD subtypes, including those with involvement of difficult-to-treat areas such as the face, neck, hands, and genital region [23]. Notably, prior exposure to dupilumab did not affect treatment response, and both upadacitinib doses demonstrated comparable efficacy, although the 30 mg dose showed a trend toward greater pruritus reduction at week 16 [23].

In terms of safety, the study reported a favorable tolerability profile. No severe infections, Herpes Zoster cases, malignancies, or MACEs occurred during the follow-up period, despite the fact that 76.3% of patients had cardiovascular risk factors and a high average Body Mass Index (BMI) [23]. AEs were generally mild, with acne flares observed in six patients and transient lymphopenia in three, none of which required treatment discontinuation. Only one patient discontinued therapy due to headaches [23]. Moreover, no cases of disease progression were reported in patients with concomitant conditions such as rheumatoid arthritis, ulcerative colitis, psoriasis, or monoclonal gammopathy of undetermined significance (MGUS), all of whom experienced stabilization or improvement of their underlying diseases [23].

In an Italian case series, seven elderly patients (median age: 72 years) with adult-onset moderate-to-severe AD were treated with upadacitinib [24]. Most had prior exposure or contraindications to cyclosporine and had not responded to dupilumab. In terms of efficacy, upadacitinib demonstrated rapid and sustained disease control across the cohort. All patients started at 30 mg/day except for two individuals who initiated treatment at the EMA-recommended 15 mg dose [24]. Most patients who began with 30 mg/day were able to reduce the dose to 15 mg/day following disease stabilization, maintaining complete or near-complete remission for up to W104 in several cases. Improvements were particularly notable in difficult-to-treat areas such as the face, hands, and genitals [24]. EASI score progressively decreased over time in all patients, as documented in follow-up visits extending to W104 in three cases [24]. The safety profile of upadacitinib in this elderly cohort was favorable. No SAEs or treatment-limiting complications occurred during the observation period [24]. Mild infections were reported in two patients—one had a skin infection treated successfully with antibiotics, and another experienced symptomatic COVID-19 that resolved without sequelae and without compromising disease control. Laboratory abnormalities were minimal: one case of mild anemia (Hb 12 g/dL) and one of pre-existing lymphopenia did not worsen during treatment [24]. Mild increases in cholesterol, triglycerides, liver enzymes, or creatine phosphokinase were observed in four patients but were clinically insignificant. Importantly, none of the patients experienced MACEs, thromboembolic complications, or malignancies during follow-up [24].

In a prospective, real-world cohort study by Tong et al., the effectiveness and safety of upadacitinib were evaluated in a group of 58 elderly patients (median age 70 years) with moderate-to-severe AD [25]. All patients were aged ≥65 years, making this one of the few studies entirely focused on the geriatric population with AD. The study specifically investigated a dose-reduction strategy guided by international treat-to-target consensus recommendations [25].

Regarding efficacy, patients exhibited rapid clinical improvement within the first month of treatment with the standard 15 mg once-daily dose. At this early timepoint, 64% of patients achieved EASI-50, and 26% reached EASI-75 [25]. Long-term control was also sustained: by month 12, 92% maintained EASI-50 and 46% reached EASI-75, despite dose tapering. The majority of patients (72%) were successfully transitioned to an every-other-day regimen, and 26% further extended to every third day, while maintaining disease control [25]. Only one patient remained on the original daily dose after one year. Importantly, no patients discontinued treatment due to inefficacy, and the mean EASI score remained low (7.94 ± 3.63) at month 12 [25]. Patient-reported outcomes reflected significant improvement, with 90% maintaining a pruritus NRS ≤4, and sustained reductions in DLQI and Patient-Oriented Eczema Measure (POEM) scores, supporting improved QoL [25].

In terms of safety, TRAEs occurred in 29.3% of patients, most of which were mild and manageable. The most common AE was herpes virus infection (13.8%), of which half required antiviral treatment, while the rest resolved spontaneously [25]. Other TRAEs included folliculitis (6.9%), gastrointestinal symptoms (5.2%), and a single upper respiratory tract infection (1.7%). Notably, two patients (3.4%) were diagnosed with VTE, identified via ultrasound after mild symptoms [25]. In total, eight patients (13.8%) discontinued treatment due to AEs, including four due to Herpes Zoster, and one each due to VTE, upper respiratory infection, and gastrointestinal issues [25]. No malignancies or deaths were reported, and no unexpected safety signals emerged throughout the 12-month follow-up [25].

#### 3.2.2. Baricitinib

A clinical observation by Hong et al. reported the outcomes of three elderly patients (aged 65, 68, and 71 years) suffering from chronic eczematous dermatitis or lichen amyloidosis, who were treated with baricitinib 4 mg/day [26]. All patients had a long-standing history of pruritus (3 to 6 years), and prior treatment attempts—including topical corticosteroids, cyclosporine, phototherapy, and dupilumab—had failed to provide sufficient control [26]. Baricitinib administration resulted in a rapid and marked clinical response, with notable improvements in both objective skin symptoms and pruritus within just two weeks. By W16 of treatment, all three patients exhibited complete resolution of pruritus, as measured by a p-NRS score reduced from 5–6 to 0 [26]. Likewise, DLQI score improved dramatically (from 10 to 0 or 1), and the POEM score normalized (from baseline scores as high as 19 down to 0–1) [26]. Importantly, the safety profile of baricitinib in this small cohort was favorable. No AEs were reported during the treatment period, which extended beyond W16 in all cases [26].

#### 3.2.3. Mixed Real-World Studies Including Multiple JAK Inhibitors

In the Italian Landscape Atopic Dermatitis (IL AD) multicenter real-life study, a cohort of 72 elderly patients (mean age 68.8 years, range 60–87) with moderate-to-severe AD was treated with one of the three approved oral JAKis: abrocitinib (N = 13; 18.1%), baricitinib (N = 6; 8.3%), or upadacitinib (N = 53; 73.6%) [27]. At baseline, patients presented with a considerable disease burden, as reflected by mean scores of 21.2 on the EASI, 18.2 on the DLQI, and 7.8 on the p-NRS [27]. Treatment with any of the three JAKis led to a rapid and significant improvement in all clinical parameters as early as W4, with continued and progressive amelioration in the following weeks for those who reached longer-term follow-up [27].

By W4, mean EASI had decreased by more than 75% (4.8), accompanied by similar reductions in DLQI (4.0) and P-NRS (1.7) [27]. These improvements were not only statistically significant but also clinically meaningful, with 66.7% of patients achieving EASI-75 and 36.1% reaching EASI-90. The response deepened over time: at W16, EASI-75 and EASI-90 response rates rose to 87.5% and 70.8%, respectively; by W24, they reached 90.9% and 81.8%, and at W52, 92.3% of patients achieved EASI-75 and 88.5% reached EASI-90 [27]. No statistically significant differences in efficacy were observed among the three JAKis, although the interpretation of comparative outcomes is limited by the small number of patients treated with baricitinib (N = 6) [27]. Moreover, patients who had previously received biologic therapy—most commonly dupilumab—showed a comparable degree of clinical improvement to those who were biologic-naïve [27]. The safety profile observed in this elderly cohort was reassuring. No patient discontinued treatment or required dose adjustment due to AEs. Overall, AEs were relatively infrequent and mostly mild, with 11 events reported across the entire cohort [27]. The most common AEs included hypercholesterolemia and nausea, each occurring in 5.6% of patients. Isolated cases of arthralgia, headache, genital herpes reactivation, leukopenia, erectile dysfunction, and myalgia were each reported in 1.4% of patients [27].

The majority of these events occurred in the upadacitinib group, that also represented the largest proportion of the treated population. Notably, no serious infections, malignancies, thromboembolic events, or cardiovascular complications were recorded, despite these being known risks associated with JAK inhibition in older adults [27]. Interestingly, certain AEs commonly observed in younger patients treated with JAKis—such as acne and upper respiratory tract infections—were not reported in this study [27].

Real-life studies on elderly patients with moderate-to-severe AD treated with JAK inhibitors have been summarized in Table 2.

## 4. Discussion

The therapeutic management of moderate-to-severe AD in elderly patients remains a clinical challenge, especially given the complex interplay of comorbidities, polypharmacy, and altered pharmacodynamics in this population. Although JAKis have emerged as promising oral options for patients with inadequate response or contraindications to biologics, their use in older adults remains cautious due to safety concerns highlighted by regulatory agencies such as the EMA [15]. In particular, increased risks of infections, malignancies, and thromboembolic events have prompted restrictive recommendations for patients aged ≥65 years [15]. Our review confirms a significant limitation in the representation of elderly patients in pivotal RCTs evaluating JAKis for AD [18]. Across studies on abrocitinib, baricitinib, and upadacitinib, elderly subjects either constituted a very small proportion of the cohort or were entirely excluded, particularly those over 75 years. Even when included, no dedicated subgroup analyses were reported, resulting in a notable gap in age-stratified efficacy and safety data [19]. This underrepresentation underscores the critical need for real-world evidence to guide clinical decision-making in this fragile subgroup. Available observational data, although still limited, provide encouraging signals. Real-life experiences with upadacitinib in elderly populations—especially those aged ≥65 years—demonstrated rapid and sustained improvements in disease severity, itch, and QoL metrics, even with dose tapering strategies [23,24,25]. Notably, dose de-escalation to alternate-day regimens in older adults did not appear to compromise disease control, suggesting potential avenues for personalized treatment schemes aimed at minimizing exposure while preserving efficacy. With regard to safety, most real-world studies described a reassuring profile in carefully selected patients. AEs were generally mild and manageable, although viral infections, particularly herpes zoster, occurred more frequently in elderly subjects [23,24,25]. This trend, especially prominent in the abrocitinib RCT subgroup analysis, highlights the importance of implementing preventive strategies such as zoster vaccination prior to treatment initiation in older patients [22]. Other safety signals—such as laboratory abnormalities (e.g., thrombocytopenia, lymphopenia), hyperlipidemia, or cardiovascular events—were more commonly reported with higher doses and in those with predisposing factors, again stressing the importance of individualized risk–benefit evaluation [23,24,25]. Interestingly, despite known concerns, cardiovascular and thromboembolic events were rarely reported in elderly cohorts treated with upadacitinib or baricitinib in real-life settings. This may reflect stringent patient selection and exclusion of individuals with significant cardiovascular history. Nevertheless, caution remains warranted, especially when initiating treatment in elderly patients with cardiovascular risk factors [23,26].

A relevant observation emerging from recent cohorts Is the absence of typical AEs reported in younger patients, such as acne, in older adults [27]. Importantly, while all three JAKis appeared effective in the elderly population, direct comparisons remain limited by heterogeneity in study design, sample size imbalance, and follow-up duration. The current literature, although promising, is insufficient to establish definitive recommendations. Future studies should aim to include elderly patients more consistently in RCTs, provide dedicated age-stratified analyses, and evaluate long-term outcomes. Prospective real-world registries, ideally with standardized AE monitoring and longer follow-up, will be essential to validate the emerging evidence base. Moreover, pharmacoeconomic analyses are needed to understand the cost-effectiveness of JAKis in the elderly, considering both direct treatment costs and indirect benefits related to disease burden reduction. In conclusion, JAKis represent a valuable therapeutic option for elderly patients with moderate-to-severe AD, particularly when conventional or biologic therapies are unsuitable. However, treatment decisions must be guided by careful patient selection, vigilant monitoring, and individualized dosing strategies. The integration of real-life data into clinical practice, combined with a growing awareness of the unique needs of older adults, will be crucial to optimizing care in this expanding patient population.

## Figures and Tables

**Table 1:** Summary of randomized clinical trials (RCTs) assessing JAK inhibitors in elderly patients with atopic dermatitis. * Safety data refer specifically to elderly subgroups when available. In trials where age-stratified safety outcomes were not reported, this is indicated accordingly.

RCT	Number of Elderly Patients	Follow-Up Duration (Weeks)	Efficacy	* Safety
Silverberg et al. Measure Up 1 and Measure Up 2 [19]	Not specified	52	No subgroup analyses were performed to assess clinical outcomes	No age-stratified safety data available
Magnolo et al. AD Up [20]	36	52	No subgroup analyses were performed to assess clinical outcomes	No age-stratified safety data available
Simpson et al. BREEZE-AD [21]	Not specified	16	No subgroup analyses were performed to assess clinical outcomes	No age-stratified safety data available
Cork et al. JADE MONO-1, MONO-2, COMPARE, REGIMEN, and EXTEND [22]	146	JADE MONO-1: 12W; JADE MONO-2: 12W; JADE COMPARE: 16W; JADE REGIMEN: 52W; JADE EXTEND: 144W	No significant differences in efficacy between elderly and overall population	**SAEs:** 19.18 vs. 15.62 per 100 PY (200 mg vs. 100 mg); **Discontinuations due to TEAEs:** 32.34 vs. 16.03 per 100 PY (200 mg vs. 100 mg) **Herpes zoster:** 10.03 vs. 5.61 per 100 PY (200 mg vs. 100 mg); **Serious infections:** 6.27 vs. 3.97 per 100 PY (200 mg vs. 100 mg) **Thrombocytopenia:** 3.17 per 100 PY (200 mg only) **Lymphopenia:** 6.29 per 100 PY (200 mg only) **NMSC:** 2.13 vs. 2.74 per 100 PY (200 mg vs. 100 mg) **Other malignancies:** 3.20 vs. 1.32 per 100 PY (200 mg vs. 100 mg) **MACEs:** 2.64 per 100 PY (100 mg only) **VTE:** 2.64 per 100 PY (100 mg only)

**Table 2:** Summary of real-world studies evaluating the effectiveness and safety of JAK inhibitors in elderly patients with moderate-to-severe atopic dermatitis.

Study	Number of Elderly Patients	Follow-Up Duration (Weeks)	Efficacy	Safety
Melgosa Ramos et al. [23]	38	16–52	65.8% achieved MDA at W16; 84.6% maintained at W52	No SAEs, HZ, MACEs, or malignancies; mild AEs (acne, lymphopenia)
Piscazzi et al. [24]	7	Up to 104	All patients responded; most maintained remission on 15 mg up to W104	No SAEs; mild infections and lab changes without clinical relevance
Tong et al. [25]	58	Up to 52	EASI-50: 92% and EASI-75: 46% at month 12	Herpes virus infection 13.8%; folliculitis 6.9%; gastrointestinal symptoms 5.2%; 13.8% discontinued; 2 VTEs; no malignancies or deaths
Hong et al. [26]	3	>16	Marked clinical response by W16; pruritus and QoL scores normalized	No AEs reported; interpretation limited by small sample size (n = 3).
Potestio et al. IL AD study [27]	72	4–52	EASI-75: 92.3%, EASI-90: 88.5% at W52; no efficacy differences between JAKis	No SAEs or discontinuations; mild AEs in 15.3%, mostly hypercholesterolemia and nausea

## Data Availability

Data are reported in the current study.
